# Membrane attack complex inhibitor CD59a protects against focal cerebral ischemia in mice

**DOI:** 10.1186/1742-2094-7-15

**Published:** 2010-03-04

**Authors:** Denise Harhausen, Uldus Khojasteh, Philip F Stahel, B Paul Morgan, Wilfried Nietfeld, Ulrich Dirnagl, George Trendelenburg

**Affiliations:** 1 Experimentelle Neurologie, Charité-Universitätsmedizin Berlin, CCM, 10117, Berlin, Germany; 2 Dept. of Orthopaedic Surgery and Dept. of Neurosurgery, Denver Health Medical Center, University of Colorado School of Medicine, 777 Bannock Street, CO, 80204 Denver, USA; 3 Department of Infection, Immunity and Biochemistry, School of Medicine, Cardiff University, Cardiff CF14 4XN, UK; 4 Max-Planck-Institute for Molecular Genetics, Ihnestr.73, 14195 Berlin, Germany

## Abstract

**Background:**

The complement system is a crucial mediator of inflammation and cell lysis after cerebral ischemia. However, there is little information about the exact contribution of the membrane attack complex (MAC) and its inhibitor-protein CD59.

**Methods:**

Transient focal cerebral ischemia was induced by middle cerebral artery occlusion (MCAO) in young male and female CD59a knockout and wild-type mice. Two models of MCAO were applied: 60 min MCAO and 48 h reperfusion, as well as 30 min MCAO and 72 h reperfusion. CD59a knockout animals were compared to wild-type animals in terms of infarct size, edema, neurological deficit, and cell death.

**Results and Discussion:**

CD59a-deficiency in male mice caused significantly increased infarct volumes and brain swelling when compared to wild-type mice at 72 h after 30 min-occlusion time, whereas no significant difference was observed after 1 h-MCAO. Moreover, CD59a-deficient mice had impaired neurological function when compared to wild-type mice after 30 min MCAO.

**Conclusion:**

We conclude that CD59a protects against ischemic brain damage, but depending on the gender and the stroke model used.

## Background

Focal cerebral ischemia leads to a primary brain damage which results from a complex pattern of pathophysiological events including excitotoxicity, periinfarct depolarizations, and inflammation [1-4]. The complement cascade is an important part of the innate immune system and is a potent mediator of inflammation and cell lysis which is activated following cerebral ischemia [5-7], and strong complement activation after ischemic stroke is associated with unfavourable outcomes [8]. Complement is deposited on apoptotic neurons which likely leads to injury in adjacent viable cells. Different studies show that blocking the complement system during the early phase of infarct evolution protects the penumbra and reduces brain injury [9,7,10]. The complement regulatory molecule CD59 represents the major controller of membrane attack complex (MAC) formation, and is an essential protector of homologous cells after complement activation [11]. CD59 is a small protein containing 10 cystein residues which form five disulfide bonds [12]. It regulates the complement activation cascade at the final step inhibiting formation of the MAC [13]. CD59 is anchored to the cell membrane via glycosyl phosphatidyl inositol (GPI), and expressed ubiquitously on cells which are in contact with body fluids containing components of the complement system including cells in the CNS. Numerous studies indicate that the MAC not only induces cell lysis but also transduces cell activation when assembled in sublytic concentrations on cell membranes [14]. For instance, the MAC has been shown to trigger the up-regulation of P-selectin and the secretion of von Willebrand factor in endothelial cells [15]. Moreover, formation of MAC was shown to trigger endothelial damage, cytotoxicity, and neurodegneration *in vivo *[16,17] and deficient expression of CD59 in a rare human disease (Paroxysmal nocturnal haemoglobinuria) is associated with an increased risk of thrombotic events [18,19]. In a model of renal Ischemia/Reperfusion (I/R), it was shown that CD59a plays a protective role in injured mice [20]. This leads to the question whether CD59a may also play a protective role in cerebral ischemia.

CD59a is constitutively expressed in neurons, most probably to protect from so-called autologous "innocent bystander" cell lysis after complement system activation in brain injury [21,22]. Nevertheless, because of low levels of neuronal CD59a expression, the neuronal capacity of controlling activation of complement is limited. This renders neurons susceptible to MAC-driven lysis in conditions of intracerebral complement activation [11]. Previous *in vitro *experiments, as well as immunostaining of human brains suggested that oligodendrocytes can also express low levels of CD59a [21]. CD59a-knockout mice [18] had a significantly impaired neurological outcome after experimental closed head injury and showed a significant exacerbation of cerebral damage when compared to wild-type controls [11].

Taken together, there is data supporting a protective effect of CD59a in cerebral ischemia which led us to the present study, in which we analysed the role of CD59a in two different standard experimental stroke models by the use of CD59a knockout mice.

## Methods

### Animals

Generation and characterization of CD59a knockout mice was described by Holt et al. (2001) [18]. CD59a-/- mice were generated on a mixed 129/Sv × C57Bl/6 genetic background and have been backcrossed to the original C57Bl/6 background for more than 10 generations. Age-matched 10 - 12 week old C57Bl/6 mice (BfR, Berlin, Germany) were used as control mice. The animal handling and surgery were performed in accordance with the *Guidelines for the Use of Animals in Neuroscience Research *(Society for Neuroscience). All experiments were approved by the local institutional Animal Care Committee, LAGeSo (No.G0382/05). The mice were bred in a selective pathogen-free (SPF) environment and under standardized conditions of temperature (21°C), humidity (60%), light and dark cycles (12:12 h), with food and water provided *ad libitum*.

### Induction of focal cerebral ischemia

Middle cerebral artery occlusion (MCAO) was induced by inserting a silicone-coated 8/0 nylon monofilament (Xantopren M Mucosa and Activator NF Optosil Xantopren, Heraeus Kulzer, Wehrheim, Germany) via the internal carotid artery as described by Hara et al. (1996) [23]. Sufficiency of occlusion and reperfusion of the middle cerebral artery (MCA) was monitored by Laser Doppler flowmetry (Peri Flux 4001 Master, Perimed, Stockholm, Sweden). Mice were anaesthetized with 2% isoflurane for induction and maintained with 1.5% isoflurane in 70% N2O and 30% O2 via a face mask. Anesthesia did not exceed 10 minutes. We used two different MCAO- models, one with a short ischemic interval (30 min) and 72 h of reperfusion [24], as well as one with a more prolonged occlusion (60 min and 48 h of reperfusion). Thirty minutes of MCAO leads to selective neuronal injury and pronounced inflammation in the striatum with little involvement of neocortical structures, while 60 min MCAO produces extensive striatal and neocortical infarction. After 30 minutes ('mild model'), or 1 h ('severe model') of ischemia the animals were re-anaesthetized and the filament was removed to permit reperfusion. During surgery and ischemia, body temperature was controlled by a temperature feedback controlled heating plate and maintained between 37.0 and 37.5°C. There was no significant difference of the mean body weight between the different groups. All experiments were performed in a randomized manner by investigators blinded to the groups as described and recommended recently by Dirnagl et al. (2009) [25].

### Neurological score

The neurologic dysfunction was determined using a neurological score (NSC) described by Bederson [26] and modified by Hara [23]. Neurological deficits were graded in wild-type and CD59a knockout mice after MCAO on a scale of: 0 - no deficit/1- failure to extend right forepaw/2 - circling to the contralateral side/3 - loss of postural reflex/4 - death. The NSC was assessed at the time-points 24 h, 48 h and 72 h after induction of cerebral ischemia for 30 min (mild model), or 1 hour (severe model). Task performance was evaluated in a blinded fashion with regard to the animal groups. Differences between wild-type and CD59a-knockout mice were analysed statistically by the Mann-Whitney U test.

### Assessment of infarct volume

Two (60 min MCAO), or three (30 min MCAO) days after induction of ischemia, mice were deeply anaesthetized and sacrificed. The brains were removed rapidly from the skull and snap frozen in 2-methylbutane on dry ice. Brains were sectioned (12 μm) on a cryostat in different coronal cryosections (positions see below), dried overnight, and stained with hematoxylin (Merck, Darmstadt, Germany). The sections were digitized, the area of infarction was quantified by using Sigma Scan Pro; Version 5.0.0 Software (Jandel Scientific, San Rafael, CA, USA), and infarct volumes were calculated. Brain swelling was calculated by subtracting the size of the whole contralateral (non-infarcted) hemisphere from the whole ispilateral (infarcted) hemisphere. Animals without an infarct or with only a small infarct in the hippocampus were excluded from these measurements because insufficient induction of cerebral ischemia was assumed. Infarct sizes of male, female, or mixed gender CD59a-deficient mice were compared to the infarct sizes of wild-type animals with matching gender. Statistical analysis was performed by using the Mann-Whitney-U-Test.

### Genotyping

Every knockout mouse used in this study was genotyped before use, which was performed using the REDExtract-N-Amp Tissue PCR kit (Sigma-Aldrich) with DNA extracted from mice tails and the following primers: Intron 3 (5'-GGT GAC CAA CTG GTG TTA ACA AAG GG-3'), neomycin '- (5'-GAA CCT GCG TGC AAT CCA TCT TG-3'), and exon 3-(5'GCT ACC ACT GTT TCC AAC CGG TG-3'). Amplification of the wild-type gene resulted in an amplicon of 212 bp, DNA derived from CD59a-ko mice produced an amplicon of 450 bp.

### Immunohistochemistry

Immunohistochemistry was performed on fresh frozen tissue harvested at different times of reperfusion. From fresh frozen tissue 12 μm coronal cryosections at interaural positions 6.6, 5.3, 3.9, 1.9, and 0 mm were thaw-mounted onto glass slides. Adjacent sections were used to determine stroke volume (see above). Slides were air-dried and fixed in -20°C methanol and acetone (1:1). The sections were incubated in blocking solution containing 3 % normal goat serum and 0.3 % Triton X-100 (Sigma). The slides were incubated 2 h at room temperature with a polyclonal rat anti-CD11b antibody (Chemicon) which stains macrophages/monocytes and microglia. For the detection of primary antibody, slides were incubated with Cy3-conjugated goat anti-rat IgG (Invitrogen) at 1:400 for 1 h at room temperature. Slides were also counterstained with Hoechst 33258, which stains DNA (Invitrogen GmbH; Karlsruhe, Germany). The whole ipsilateral hemispheres of three sections each animal (n = 3) were counted using stereo investigator 7 (MicroBrightField Bioscience, Williston, USA).

### Terminal deoxynucleotidyl transferase dUTP nick end labeling (TUNEL)

The Fluorescein in Situ Cell Death Detection Kit (Roche Diagnostics GmbH, Mannheim, Germany) was used for TUNEL stain. Adjacent slides for immunohistochemical staining (see above) were used for the detection of damaged cells. Slides were dried and fixed in 4% formalin solution. After washing, the sections were incubated in ice-cold ethanol-acetic acid solution (3:1) for 10 min followed by incubation for 1 h in 3% Triton-X 100 (Sigma-Aldrich). Next, sections were incubated with TdT-enzyme in reaction buffer containing fluorescein-dUTP for 90 min at 37°C. Enzyme was omitted for the negative control. After washing, slides were also counterstained with Hoechst 33258 (Invitrogen GmbH; Karlsruhe, Germany). All sections were evaluated after staining using Leica stereo investigator 7 (MicroBrightField Bioscience, Williston, USA).

### Statistical analysis

For comparison of infarct volumes and neurological deficit Mann-Whitney-U-test was used, if not stated otherwise. P-values below 0.05 were considered statistically significant. Power calculation was performed using SISA-Binominal [27]. Based on the known variance of previous experiments the MCAO experiments were powered (α = 0.05; β = 0.8) to detect effect sizes *d *[28] of at least 1, i.e. of one standard deviation.

## Results

### CD59a-deficiency increases infarct volume in mild experimental stroke, but not in the more severe stroke model

First, we analysed whether CD59a-deficiency alters the size of the infarcted area in two different models of cerebral ischemia: a more severe stroke model (60 min occlusion time) and a mild one (30 min occlusion time).

**Figure 1:**
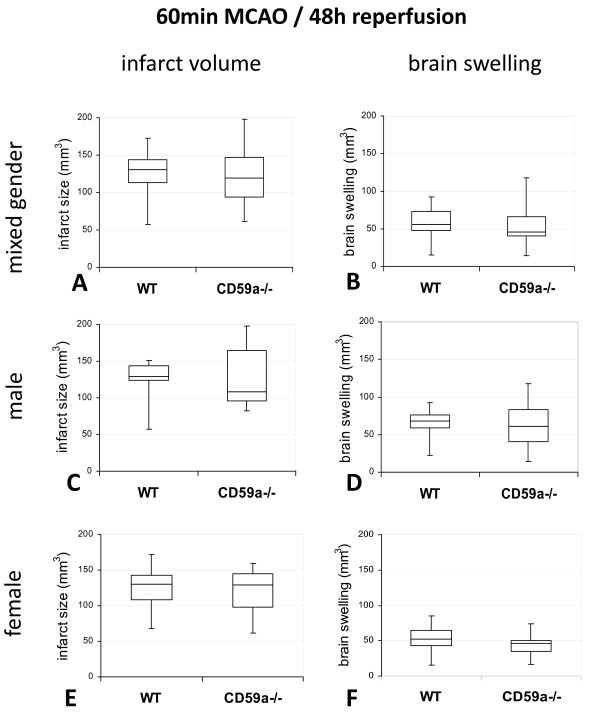
**Comparison of infarct volume and brain swelling of CD59a-deficient and wild-type-mice (C57BL/6) after 60 min MCAO**. Infarct volumes (**A, C, E**), and brain swelling (**B, D, F**), given as box-and-whisker plots, at 48 h of reperfusion after induction of ischemia are visualized for both, CD59a-deficient (**CD59a-/-**) and wild-type (**WT**) mice, of both genders (**A, B**) (n = 22_CD59a-/- mix_; n = 16_WT mix_) as well as male (**C**, **D**) (n_CD59a-/- m _= 11; n_WT m _= 6) and female (**E, F**) (n_CD59a-/- f _= 11; n_WT f _= 10) mice separately. Statistical analysis was performed using the *Mann-Whitney-U*-test. The indirect infarct volume was calculated as the volume of the difference between contralateral hemisphere and the non-infarcted volume of the ipsilateral hemisphere. There is no significant difference between infarct sizes of CD59-ko mice and wild-type mice. In all box plots, the top of the box represents the 75th percentile, the bottom of the box represents the 25th percentile, and the line in the middle represents the 50th percentile (median). The whiskers (the lines that extend out the top and bottom of the box) represent the highest and lowest values that are not outliers or extreme values.

When infarct volumes of male and female CD59a-deficient mice and matching wild-type mice were compared after 1 h MCAO and 48 h of reperfusion, no significant difference between CD59a-knockout mice and wild-type mice, either in males (values are given as median [25^th ^percentile, 75^th ^percentile]: CD59a_m_-/-: 108 mm^3 ^[96 mm^3^, 164 mm^3^]; WT_m_: 129 mm^3 ^[123 mm^3^, 144 mm^3^], in females (CD59a_f_-/-: 129 mm^3 ^[98 mm^3^, 145 mm^3^] WT_f_: 130 mm^3 ^[109 mm^3^, 143 mm^3^], or in the mixed-gender group was detected (CD59a_mix_-/-: 119 mm^3 ^[94 mm^3^, 147 mm^3^] WT_mix_: 130 mm^3 ^[113 mm^3^, 144 mm^3^]) (Figure 1A,C,E). There was also no significant difference of brain swelling after 60 min MCAO and 48 h reperfusion between CD59a-knockout and wild-type mice of either gender (median: CD59a_mix_-/-: 46 mm^3^; WT_mix_: 56 mm^3^) (Figure 1B,D,F). For Type II error considerations, see *Methods *(Statistics).

**Figure 2:**
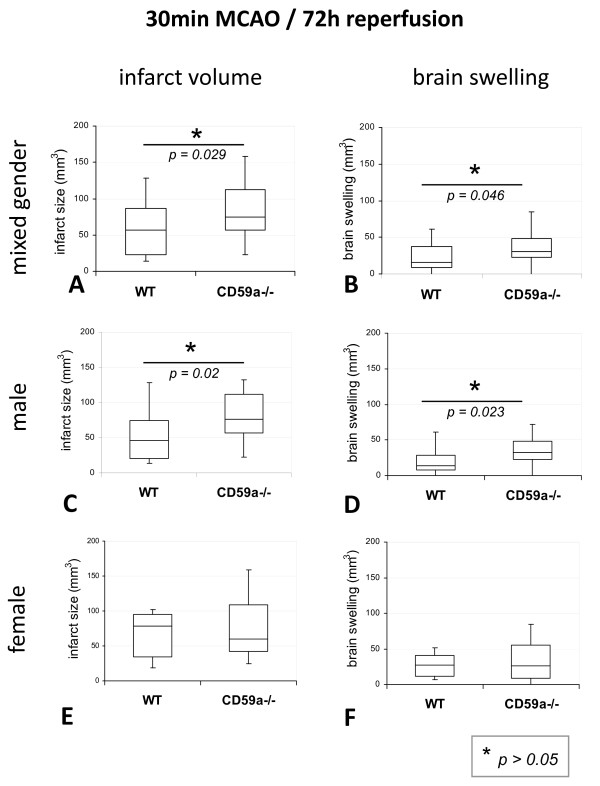
**Comparison of infarct volume and brain swelling of CD59a-deficient and wild-type-mice (C57BL/6) after 30 min MCAO**. Infarct volumes (**A, C, E**), as well as volume of brain swelling (**B, D, F**) given as box-and-whisker-plots are visualized. Statistical analysis was performed with the *Mann-Whitney-U-*test (*: p ≤ 0.05). In contrast to 1 h MCAO-model (figure 1), after 30 min MCAO and 72 h of reperfusion there was a significant difference of infarct volumes and brain swelling between wild-type and CD59a-deficient mice in both, mixed gender and male mice: infarct volumes were larger and brain swelling increased in male CD59a knockout mice (n_CD59a-/- m _= 22; n_WT m _= 18), as well as in knockout mice of mixed gender (n = 25_CD59a-/- mix_; n = 24_WT mix_), when compared to age- and gender-matched wild-type control mice. No significant differences were seen for female mice. In all box plots, the top of the box represents the 75th percentile, the bottom of the box represents the 25th percentile, and the line in the middle represents the 50th percentile. The whiskers (the lines that extend out the top and bottom of the box) represent the highest and lowest values that are not outliers or extreme values.

However, when mice were subjected to the second stroke model (30 min MCAO), a statistically significant increase of the infarct volume was observed in male CD59a-knockout mice and in CD59a-knockout mice of mixed gender when compared to wild-type mice at 72 h of reperfusion (difference of the infarct volume in the mixed gender group, given as median [25^th ^percentile, 75^th ^percentile]: CD59a_mix_-/-: 74 mm^3 ^[57 mm^3^, 113 mm^3^]; WT_mix_: 57 mm^3 ^[23 mm^3^, 87 mm^3^]; p = 0.029) (Figure 2A). The difference was statistically significant only in male mice (infarct volume in male mice: CD59a_m_-/- 77 mm^3 ^[57 mm^3^, 112 mm^3^] vs. WT_m_: 46 mm^3 ^[20 mm^3^, 74 mm^3^] p = 0.020; infarct volumes in female mice: CD59a_f_-/- 60 mm^3 ^[42 mm^3^, 109 mm^3^] vs. WT_f_: 78 mm^3 ^[34 mm^3^, 95 mm^3^]) (Figure 2C,E). Brain swelling correlated with infarct volumes, because brain swelling - as calculated by the size difference of the ischemic and nonischemic hemisphere - was significantly increased in CD59a-/- male mice (median: CD59a_m_-/-: 33 mm^3^; WT_m_: 14 mm^3^) and mixed gender (median: CD59a_mix_-/-: 31 mm^3^; WT_mix_: 16 mm^3^), but not female mice (Figure 2B,D,F).

### CD59a-deficiency leads to increased apoptosis only in the more severe experimental stroke

**Figure 3:**
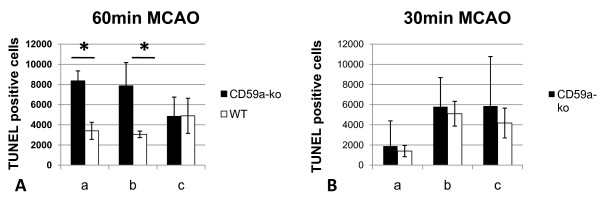
**TUNEL-positive cell count in post-ischemic mouse brain tissue after 30 min and 60 min MCAO**. Absolute counts of TUNEL (apoptotic and necrotic)-positive cells in whole ischemic brain hemispheres of male wild-type and CD59a-deficient mice, determined in different brain sections. **A**: TUNEL-positive cell counts in MCAO-model 1 (60 min MCAO), **B**: cell counts in MCAO-model 2 (30 min MCAO). Distance to bregma: **a**, 5.3 mm; **b**, 3.9 mm; and **c**, 1.9 mm (*: p < 0.05, SD, unpaired t-test, n = 3 per group).

**Figure 4:**
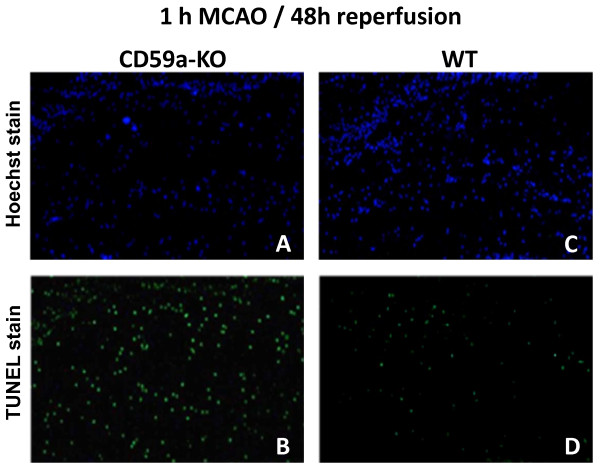
**Representative images of TUNEL-positive cells in post-ischemic mouse brain tissue after 60 min MCAO and 48 h reperfusion**. TUNEL (apoptotic and necrotic)-positive cells (**B**, **D**) in ischemic brain tissue of the infarct border zone (penumbra) of male wild-type (**C**, **D**) and CD59a-deficient mice (**A**, **B**). **A**,**C**: staining of cell nuclei using Hoechst 33258, which stains DNA (Invitrogen, Germany).

Next, we compared the amount of TUNEL-positive cells in CD59a-deficient and wild-type mice in both models, because we speculated that MAC-inhibition by CD59a may influence the amount of apoptotic cell death, which depends on the stroke model used [24,29]. TUNEL-positive cells were mostly found in the infarct border zone ('penumbra'), the extend and location of which depends on the stroke model used: e.g. compare amount of TUNEL-positive cells in the different sections of both models (Figure 3A,B). Indeed, there was a significant increase of TUNEL-positive cells found in the ischemic hemisphere of male CD59a-deficient mice at 48 h after 60 min MCAO when compared to wild-type controls (Figure 3 and 4). Since infarct volumes did not differ significantly, this observation argues against a pure 'secondary effect', meaning that the amount of apoptotic cells only depends on the size of the infarct volume. On the other hand, the amount of TUNEL-positive cells did not differ significantly between CD59a-deficient and wild-type mice when the mild stroke model (30 min MCAO/72 h reperfusion) was used (Figure 3).

### CD59 deficiency has no influence on CD11b-positive cell accumulation in post-ischemic brain tissue

Because sublytic MAC deposition was recently shown to be associated with the release of proinflammatory cytokines (e.g. TNFa) and chemotactic factors (IL-8) [15,30], the post-ischemic inflammatory response was determined in CD59a-deficient mice and wild-type mice by counting CD11b-positive cells in the ischemic hemisphere. Staining with CD11b, which stains invading macrophages/monocytes as well as activated microglia, revealed that there was no significant alteration of CD11b-positive cells in the ischemic hemisphere of CD59a-ko mice (section 1: 301 ± 4 [60 min MCAO], 252 ± 61 [30 min MCAO]; section 2: 410 ± 123 [60 min MCAO], 365 ± 96 [30 min MCAO]); section 3: 600 ± 342 [60 min MCAO], 188 ± 52 [30 min MCAO]) when compared to that of wild-type mice (section 1: 337 ± 110 [60 min MCAO], 162 ± 102 [30 min MCAO]; section 2: 530 ± 197 [60 min MCAO], 437 ± 34 [30 min MCAO]; section 3: 575 ± 286 [60 min MCAO], 259 ± 102 [30 min MCAO]) in both models (48 h after 60 min MCAO; 72 h after 30 min MCAO) (data not shown).

### CD59a-deficient mice have a worse neurological deficit when compared to wild-type mice after 30 min MCAO

To investigate if CD59a-deficiency also influences the neurological outcome after cerebral ischemia, CD59a-knockout as well as wild-type control mice of mixed gender were subjected to experimental stroke with two different occlusion times (MCAO for 30 min, respectively 60 min). The neurological deficit was determined at different times of reperfusion after induction of cerebral ischemia.

**Figure 5:**
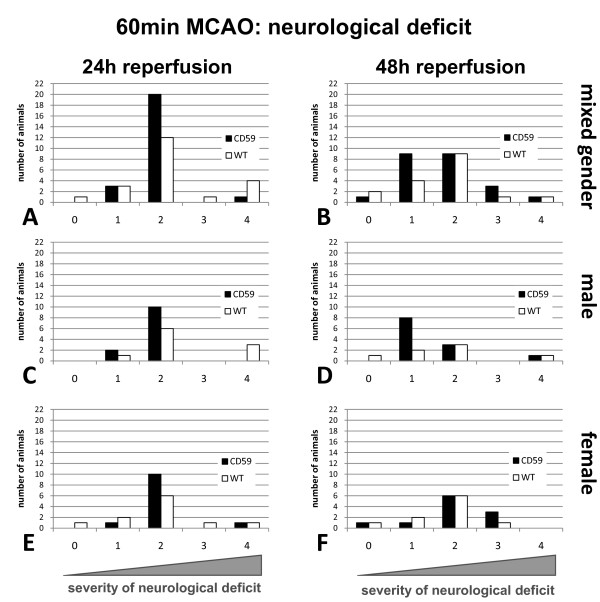
**Comparison of neurological dysfunction of CD59a-deficient and wild-type-mice (C57BL/6) after 60 min MCAO**. Either 24 h after reperfusion (**A, C, E**) or 48 h after reperfusion (**B, D, F**) there is no significant difference in neurological dysfunction, neither in male (**C**, **D**) nor in female (**E**, **F**) mice group when comparing CD59-deficient mice to wild-type (WT) mice. Statistical analysis was performed using the *Mann-Whitney-U*-test. Score of 0: no neurological dysfunctions; score 1: failure to extend right forepaw, score 2: circling to the contralateral side; score 3: loss of postural reflex and score 4: death. Number of animals: for CD59a-ko n = 24, WT n = 21. Both male and female animals were used in this study.

Neither at 24 h nor at 48 h reperfusion time was there a significant difference of the neurological deficit between CD59a-ko (n = 24) and wild-type mice (n = 21) when middle cerebral artery was occluded for 60 min (Figure 5).

**Figure 6:**
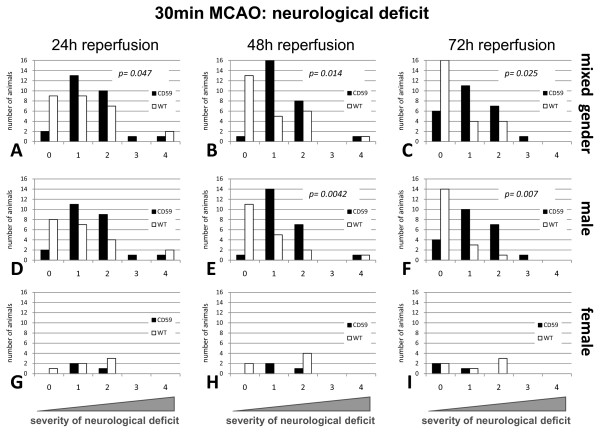
**Comparison of neurological dysfunction of CD59a-deficient and wild-type-mice (C57BL/6) after 30 min MCAO**. The CD59-ko mice in the mixed gender group show 24 h after reperfusion (**A**), 48 h after reperfusion (**B**) and 72 h after reperfusion (**C**) a significant increased neurological dysfunction (p < 0,05) compared to wild-type (WT) mice. In the male gender group (**D, E, F**) there is only a significant difference after 48 h (**E**) and 72 h (**F**). No increase was observable in the female group (**G, H, I**). Statistical analysis was performed using the *Mann-Whitney-U*-test. A score of 0 shows no neurological dysfunctions; score 1: failure to extend right forepaw, score 2: circling to the contralateral side; score 3: loss of postural reflex and score 4: death. Number of animals: for CD59a-ko n = 27, WT n = 27. Both male and female animals were used in this study.

However, in contrast to the findings with the more severe stroke model, a significant neurological deterioration (p < 0.05) was observed after 30 min occlusion of the middle cerebral artery (mild stroke model) in CD59a-deficient mice of mixed gender and male gender when compared to the wild-type mice. At all three time points examined (24 h, 48 h, and 72 h after induction of MCAO), there was a significant increase in the neurological deficit (p_24 h _= 0.047; p_48 h _= 0.014; p_72 h _= 0.025 as calculated by Mann-Whitney U test) in CD59a-deficient mice (n = 27) when compared to wild-type control mice (n = 27). Male mice showed only a significant difference after 48 h and 72 h (p_48 h _= 0.0042; p_72 h _= 0.007) (Figure 6).

## Discussion

This study demonstrates that deficiency of the complement regulator protein CD59a exacerbates post-ischemic brain damage *in vivo *in a gender-specific way and depending on the severity of the stroke.

The complement system has been recently identified as a conductor of inflammation triggering further tissue damage in experimental ischemia [31]. Activation of the complement system mediates secondary brain injury, which leads to increased infarct volumes, a pronounced inflammatory response, and a worse neurological deficit [32,33]. Recent studies indicated that both, the classical and the mannose-binding lectin (MBL) complement pathways trigger complement activation [6,34,35], but the alternative pathway also appears to play a crucial role in neuronal death [36,37]. Complement activation results in the release of chemotactic factors (e.g. C3a, C5a) and finally in the formation of the terminal pore-forming complex, the membrane attack complex (MAC), by complement components C5b-9. Formation of sublytic concentrations of the MAC was shown to induce the release of several pro-inflammatory cytokines (e.g., TNF-α) and chemotactic factors including IL-8 and monocyte chemoattractant protein (MCP)-1 [15,38]. However, the exact contribution of the MAC to the complement-mediated injury in cerebral ischemia has remained unclear. The complement regulator protein CD59 is known to inhibit MAC formation by preventing the incorporation and polymerization of C9 on cell membranes [13]. CD59a has been characterized as the primary regulator of MAC assembly in the mouse, since the expression of the second CD59 isoform in mice, CD59b, was found to be restricted to testis [39].

Thus, we used CD59a-deficient mice to study the role of MAC and its inhibitory protein CD59a in focal cerebral ischemia. Our data reveal that the presence of CD59a improves neurological outcome, decreases brain swelling, and reduces infarct volume after 30 min of transient focal cerebral ischemia in the mouse. We have previously shown an increased neurological deficit in CD59a-knockout mice when compared to wild-type mice in a model of traumatic brain injury [11]. Moreover, a stroke study in neonatal rats, naturally lacking C9 - which is part of the terminal MAC - demonstrated an increase of infarct volumes in neonatal rats when purified C9 was injected [40]. Further, CD59 was shown to efficiently protect human NT2-N neuronal cells against complement-mediated cell injury [41,42].

Our data revealed that significant protection by the presence of CD59a is gender-specific and is only achieved in male mice. This difference might be explained by the influence of hormonal fluctuations in female mice. Gender-specific neuroprotective effects have been also found by others [43-45]. A substantial amount of data indicates that progesterone, a gonadal hormone and neurosteroid naturally distributed in the human brain, has potent neuroprotective properties [46,47]. In animal studies, progesterone reduced cerebral edema, neuronal loss and behavioral deficits by inhibiting secondary injury cascade [48-52]. Safety and efficacy phase I and II trials have been succesfully conducted in recent clinical trials [53,54]. It appears that the gender-specific neuroprotective effects seen in the present study may be related to progesterone-mediated beneficial effects in female animals.

Moreover, the degree of protection by CD59a depends on the stroke model used and was only observed in the stroke model with the more selected neuronal death (mild stroke model). This observation fits well with the data of Yamada et al. [55], which observed only a synergistic effect of CD59a deficiency (together with CD55) in renal ischemia. The prolonged occlusion interval (60 min MCAO) leads to a significant increase of TUNEL-positive cells in the CD59a-deficient mice when compared to wild-type mice, which is in good agreement with the findings of Turnberg et al. [20], who obtained similar results using CD59a-/- mice in a model of renal ischemia.

It was recently shown that ischemia leads to a rapid loss of membranous CD59 protein in the ischemic core region [41,6]. Thus, we alternatively speculate that the longer occlusion interval in the 60 min MCAO model may dampen local CD59a-expression, so that the remaining CD59a expression in the wild-type mice (CD59a+/+) is of minor relevance when compared to the complete lack of CD59a expression in the knockout mice. An alternative explanation may be that both models (30 min vs. 60 min occlusion interval) are characterized by different degrees of complement activation: in the postischemic brain tissue of the 30 min MCAO model complement activation may only result in sublytic MAC-concentrations, so that the MAC-mediated effects not directly result in cell lysis (as it may be the case in the 60 min MCAO model), but rather contribute to the secondary brain injury by the induction of proinflammatory chemokines, which renders the postischemic damage more dependent on MAC-inhibition. We postulate that CD59a-deficiency mainly contributes to postischemic neuronal damage by increasing MAC-induced neuronal cell death, but that this effect is further increased by the modulation of the innate immune system (invading leukoycytes as well as activated glia) as reviewed by Griffiths et al. (2009) [56]. Nevertheless, the different reperfusion intervals in both models used do not allow a direct comparison of infarct outcomes. Accordingly, effects of CD59a-deficiency differ significantly in renal ischemia, depending on the time point used for evaluation [20,55].

MAC formation was recently shown to trigger up-regulation of pro-inflammatory cytokines, chemotactic factors, as well as adhesion molecules such as P-Selectin, E-selectin and ICAM-1 in activated endothelial cells [15,30,38]. Nevertheless, we did not detect a significant increase of the inflammatory cell accumulation in the CD59a-deficient mice when compared to wild-type mice. This observation may be due to the more prominent effect of other chemoattractant proteins, which were upstream of the MAC in the complement cascade (e.g. C3a, C5a), and which were not affected by CD59a expression. An alternative explanation may be that the part of the inflammatory response which is based on the MAC-driven induction of pro-inflammatory genes is not detected at the time points studied in our setting and may be more relevant at earlier time points as observed by [57].

Nevertheless, our study is in good agreement with various reports, that show an improved neurological function after experimental stroke in animals treated with complement-inhibitors [33], treated with the complement-depleting agent cobra venom factor [58,9], or in animals which lack complement components, e.g. C3-deficient mice [59]. The ongoing debate of whether the complement system is 'friend or foe' in ischemic brain injury [60] may be explained by the complexity of the system and the manifold pathways which are activated by the complement system, and the different ways complement may be inhibited at different levels of the complement cascade. Thus, our data which demonstrates model- and gender-specific effects of MAC-inhibition by CD59a, replenishes the current understanding of the complement system in ischemic brain injury and thus may contribute to the development of future therapeutical strategies [61].

## Conclusions

Based on the data, we conclude that the complement inhibitor protein CD59 is protective after cerebral ischemia in a gender specific way, and that this effect depends on the severity of the cerebral damage. In a mild model of cerebral ischemia with selective neuronal cell death, CD59 leads to less neuronal dysfunction and a smaller infarct volume. However, the exact mechanisms of complement MAC-induced neuronal cell death after cerebral ischemia require further investigation.

## Competing interests

The authors declare that they have no competing interests.

## Authors' contributions

DH designed the experiments, performed all experiments, analysed the data, generated the figures, and wrote the manuscript. UK did parts of the animal surgery and revised the manuscript. PFS, BPM, and WN participated in the experimental design, and in the editing of the manuscript. UD provided overall study supervision and intellectual input. GT participated in the experimental design and preparation of the manuscript. All authors have read and approved the manuscript.
